# Factors related to the risk of stroke in the population with type 2 diabetes: A protocol for systematic review and meta-analysis

**DOI:** 10.1097/MD.0000000000027770

**Published:** 2022-01-21

**Authors:** Kai Wang, Zhiguo Lv, Peng Xu, Yabin Cui, Xiaoyu Zang, DongMei Zhang, Jian Wang

**Affiliations:** a Changchun University of Chinese Medicine, 1035 Boshuo Road, Changchun City, Jilin Province, China; b Affiliated Hospital of Changchun University of Chinese Medicine, 1478 Gongnong Road, Changchun City, Jilin Province, China.

**Keywords:** meta-analysis, risk factor, stroke, systematic review, type 2 diabetes

## Abstract

**Background:**

Type 2 diabetes is an independent risk factor for stroke. The main role of the current study is to study the mechanism of stroke induced by diabetes, but there is no systematic summary of daily management and stroke prevention for patients with type 2 diabetes. In order to provide a more detailed stroke prevention program for patients with type 2 diabetes, we included in the study and looked forward to analyzing the risk factors that were more in line with the clinical characteristics of type 2 diabetes.

**Methods:**

We will search the following Chinese and English databases: PubMed, Web of science, Cochrane Library, Medline, and China National Knowledge Infrastructure database. All of the above electronic databases will be searched from inception to June 30, 2021. In addition, we will manually search for conference papers, ongoing experiments, and internal reports to supplement the studies retrieved via electronic search. We will use the STATA 16.0 provided by Cochrane Collaboration Network for statistical analysis.

**Results:**

The study will prove a collective view on the relationship between related factors and stroke in the type 2 diabetes population.

**Conclusion:**

We plan to submit this systematic review to a peer-reviewed journal.

**INPLASY registration number:** INPLASY2021100046

## 1 Introduction

Type 2 diabetes (T2D) is a global pandemic, with an estimated 370 million people currently affected.^[1,2]^ Stroke is an independent disorder and a typical macrovascular complication of T2D, and it is regarded as the second leading cause of death after ischaemic heart disease.^[3,4]^ Diabetes mellitus, characterized by chronic hyperglycaemia due to an absolute or relative deficiency in insulin, is a major risk factor for stroke. People with diabetes are at a 2 folds to 5 folds increased risk for stroke compared with people without diabetes.^[5–9]^ It is necessary to further understand the factors related to stroke risk in people with type 2 diabetes. Over the past decade, a number of prediction models (or risk scores) have been developed incorporating these risk factors to predict a person's risk of developing stroke. Risk factors for stroke include lifestyle-related factors, predisposing medical conditions, specific genetic diseases, as well as sociodemographic factors.^[10–12]^ Their research shows that the risk prediction model composed of these related factors has a considerable predictive and early warning effect on stroke in the diabetic population. Some recent studies have revealed the safety results of patients with chronic kidney disease and high vascular risk in diabetes medications, and the renal mechanism of action may have a weakening effect.^[13]^ In the collaborative analysis of 12 Asian cohorts, a 27% increase in risk of hemorrhagic stroke was shown for a 0.6 mmol/L decrease in cholesterol concentrations.^[14]^ Considering the special population of T2D, the stroke-related factors we explored mainly include general information, medical history, and clinical biochemical indicators. These related factors that are close to the population are better equipped. We hope that the results of this study can have an impact on the structure of stroke prediction and warning for people with T2D, and effectively prevent the occurrence of stroke.

## 2 Methods and analysis

### 2.1 Information sources and search strategies

According to the Cochrane Handbook, we systematically searched studies reporting the effect of related factors on type 2 diabetes in the PubMed, Web of Science, Cochrane Library, Medline, and China National Knowledge Infrastructure database. And through the meta-analysis method to get the exact effect size. We combined search terms describing the exposure with each outcome of interest. The following mesh terms were used: “Type 2 diabetes,” “Stroke,” “Risk factors,” and “Relevant factor.” Furthermore, a manual search was carried out from all references cited in original studies and in all reviews identified. The prospective registration has been approved by the International Platform of Registered Systematic Review and Meta-analysis Protocols (https://inplasy.com/inplasy-2021-10-0046/) under registration number inplasy2021100046.

### 2.2 Inclusion criteria

Eligible studies must meet the following criteria: prospective cohort study, nested case-control study; have clear diagnostic criteria; exclude reviews, comments, case reports, animal experiments; OR and 95% confidence interval (CI) (or calculated result data) of stroke-related factors in the T2D population must be reported; Or the OR and 95% CI of stroke per change unit related factors must be given. After omitting duplicate studies and/or studies in the same cohort, the most complete and recent study were finally included.

### 2.3 Exclusion criteria

The exclusion criteria will be as follows: studies that do not meet the above inclusion criteria; incomplete or incorrectly researched data; case reports, comments or letters, biochemical tests, protocols, meeting abstracts and reviews.

### 2.4 Outcome measures

The main outcome was all risk factors for stroke in type 2 diabetic patients.

### 2.5 Literature screening and data extraction

#### 2.5.1 Literature screening

Two researchers used standard data extraction tables to collect the following information: article metadata, including the name of the first author; publication year, country or region, sample size, number of stroke cases, and related factors reported. We extracted the OR and 95% CI of stroke in the T2D population for follow-up analysis. Choose the most appropriately adjusted model to evaluate the risk value of the final analysis when extracting the data. If the study does not report a risk estimate, an unadjusted estimate will be calculated based on the number of cases and controls in the defined category of related factors.

Literature screening process: Endnote software will be used to eliminate duplicate literature; the title and abstract of the articles will be read, and irrelevant literature will be excluded; the full text of the remaining articles will be read to determine whether these studies will be included in the study.

#### 2.5.2 Data extraction

The following information will be extracted: research characteristics, including title, first author, research year and country; basic characteristics of the research object, including number of cases, intervention measures, treatment types, intervention time, outcome indicators, etc; key elements of bias risk assessment; and research results.

### 2.6 Assess the risk of bias in the included studies

Two researchers will use the Newcastle-Ottawa Scale was used to assess the quality of all included studies. The scale has 3 evaluations. The selection of the case group and the control group, the comparability of the case group and the control group, the exposure assessment method, the total score is 10 points. Studies with a Newcastle-Ottawa Scale score of 5 and above are considered high-quality studies and are eligible for inclusion in this meta-analysis.

### 2.7 Assessment of heterogeneity and reporting bias

We will use the standard *I*^2^ test to evaluate statistical heterogeneity; *I*^2^ < 50% indicates that there is no significant heterogeneity, and *I*^2^ ≥ 50% indicates significant heterogeneity. We will use a funnel chart to assess publication bias.

### 2.8 Statistical analysis of data

We will use STAT 16.0 software for the meta-analysis. We will use relative risk as an effective indicator of counting data and use the mean differences as an effective indicator of measurement data. The confidence interval for each effect index will be set to 95%. Additionally, *I*^2^ will be used to quantitatively assess heterogeneity. If there is no statistical heterogeneity between the studies, a fixed effects model will be used for the meta-analysis. If there is heterogeneity, a random effects model will be used. *P* < .05 indicates statistical significance.

### 2.9 Analysis of subgroups or subsets

When there is potential heterogeneity in this study, if all the information included in the study was available, we could perform subgroup analysis based on the sex, age, and treatment time of the included subjects.

### 2.10 Sensitivity analysis

A sensitivity analysis will be performed to assess the robustness of the included results. If the results are unstable, studies with a high risk of bias will be excluded.

### 2.11 Ethics and publishing

These data are obtained from the database and do not require ethical approval. We will submit the final research results to a peer-reviewed journal for publication.

## 3 Discussion

One study found that the large fluctuation of blood glucose can cause oxidative stress, accelerate apoptosis of endothelial cells, damage various target organs, decrease of tissue plasminogen activator and type I fibrinolytic enzyme, and cause or aggravate the occurrence of stroke.^[15]^ Diabetes can cause macrovascular disease. Ischemic and small vessel disease increased significantly in all ages risk of stroke.^[16]^ T2D has become the main cause of stroke, and glycosylated hemoglobin level is an independent risk factor for stroke. However, obesity, exercise, blood sugar fluctuation, mood, and other common risk factors for stroke in patients with type two diabetes have not been assessed in detail. The object of this study is to systematically study the risk factors of stroke in patients with T2D, and find new and more accurate and reliable evidence for providing theoretical guidance for the prevention and treatment of stroke, early screening and health education, and standardize the follow-up management of diabetic patients, early detection of high-risk groups, and timely intervention to reduce the risk of stroke.

## Author contributions

**Conceptualization:** Kai Wang.

**Data curation:** Kai Wang, Xiaoyu Zang, Zhiguo Lv.

**Formal analysis:** Kai Wang, Peng Xu, Yabin Cui.

**Funding acquisition:** Jian Wang, DongMei Zhang.

**Investigation:** Peng Xu, Kai Wang.

**Methodology:** Zhiguo Lv.

**Project administration:** DongMei Zhang.

**Resources:** Yabin Cui.

**Software:** Kai Wang, Xiaoyu Zang, Zhiguo Lv.

**Supervision:** Jian Wang, DongMei Zhang.

**Visualization:** Peng Xu, Kai Wang, Yabin Cui.

**Validation:** Kai Wang, Xiaoyu Zang.

**Writing – original draft:** Kai Wang.

**Writing – review & editing:** Kai Wang, Jian Wang, DongMei Zhang.
